# Efficacy evaluation of contrast-enhanced magnetic resonance imaging in differentiating glioma from metastatic tumor of the brain and exploration of its association with patients’ neurological function

**DOI:** 10.3389/fnbeh.2022.957795

**Published:** 2022-09-06

**Authors:** Zhuo Shi, Jiuming Jiang, Lizhi Xie, Xinming Zhao

**Affiliations:** ^1^Department of Imaging Diagnosis, National Cancer Center/National Clinical Research Center for Cancer/Cancer Hospital, Chinese Academy of Medical Sciences and Peking Union Medical College, Beijing, China; ^2^GE Healthcare, MR Research China, Beijing, China

**Keywords:** MRI, glioma, metastatic tumor of brain, apparent diffusion coefficient, fractional anisotropy, K^trans^

## Abstract

**Objective:**

To determine the efficacy of contrast-enhanced MRI in differentiating glioma (GL) from the metastatic tumor of the brain (MTB) and its association with patients’ neurological function.

**Methods:**

A retrospective analysis was conducted on 49 cases of pathologically confirmed GL and 42 cases of MTB admitted between April 2019 and January 2022. All patients were examined by a set of MRI sequences that included T1WI, T2WI, FLAIR, and DWI. The values of fractional anisotropy (FA), apparent diffusion coefficient (ADC), and operation coefficient (K^trans^) were calculated by taking the tumor parenchyma area, cystic area, and peritumor edema area as the regions of interest (ROIs). And according to the Mini-mental state examination (MMSE) results, the contrast-enhanced MRI with patients’ neurological dysfunction was observed.

**Results:**

The clinical symptoms and MRI findings of MTB and GL were basically the same, mainly showing neurological symptoms. The tumor parenchyma area and cystic area were mainly located in the tumor periphery and tumor central area, respectively, while the peritumor edema area was widely distributed, showing an irregular patchy edema zone. Contrast-enhanced scans suggested an obvious enhancement in the tumor parenchymal area, presenting with nodular and annular enhancement, but no enhancement in the tumor cystic and peritumor edema areas. There was no difference between GL and MTB in FA values of tumor cystic area and peritumor edema area (*P* > 0.05), but the FA value of the parenchyma area of GL was higher (*P* < 0.05). Besides, GL and MTB showed no difference in ADC and K^trans^ values (*P* > 0.05), while the former presented lower ADC values and higher K^trans^ values of the peritumor edema area than the latter (*P* < 0.05). In patients with GL and MTB, the FA and K^trans^ values of all ROIs in those with neurological dysfunction were higher compared with those without neurological dysfunction, while the ADC values were lower (*P* < 0.05).

**Conclusion:**

Contrast-enhanced MRI of peritumor edema area can effectively distinguish GL from MTB, and improve the accuracy of early clinical screening, thus providing more reliable life security for patients.

## Introduction

Glioma (GL), a collective term for tumors derived from keratinocytes and neurons of the nervous system, is also the most common intracranial malignant tumor, accounting for more than 40% of all intracranial neoplasms (Xu et al., 2020). At present, the global incidence of GL is about 3–8/100,000, and the incidence is increasing trend. Therefore, the disease has become a global public health problem, causing a great burden to the clinic (Gusyatiner and Hegi, 2018). Moreover, GL, as one of the tumors with high malignancy, is basically asymptomatic at the initial stage except for dizziness and headache in some cases. However, when the patient begins to develop epilepsy, and visual and motor disorders, the disease has developed to the middle and late stage, which further increases the potential threat and the treatment difficulty (Nicholson and Fine, 2021). According to statistics, more than one-third of patients with GL have a disease that progressed to high-grade GL at the time of diagnosis, and the 3-year mortality rate exceeds 60% (Wang and Mehta, 2019). Therefore, how effectively and quickly make an accurate diagnosis in the early stage of GL is the key to ensuring the life safety of patients with GL.

At this stage, the early clinical evaluation of neoplastic diseases is still primarily based on imaging technology. Among them, magnetic resonance imaging (MRI) is characterized by various imaging methods such as diffusion tensor imaging, fully weighted imaging, and susceptibility-weighted imaging, and is one of the most commonly used methods, which can show the manifestation of tumor diseases from multiple aspects (Gannon et al., 2019; Smits, 2021). In GL, the evaluation significance of MRI, which has been repeatedly verified in a number of studies, is also worthy of recognition (Almansory and Fraioli, 2019; Hu et al., 2020). However, metastatic tumors of the brain (MTB) and GL have very similar presentations and can easily lead to misdiagnosis, which is the most important differential diagnosis in the MRI evaluation of GL (Priya et al., 2021). Although there have been previous guidelines for the evaluation of GL or MTB by contrast-enhanced MRI (Gao et al., 2016; Abecassis et al., 2020), there is still a lack of reliable research demonstration on the specific distinction criteria between the two, leaving a great controversy in clinical practice.

In view of the above, this study analyzes the effect of MRI in differentiating GL from MTB, and further explores the correlation of contrast-enhanced MRI with patients’ neurological function, so as to provide reliable reference and guidance for future clinical differentiation between GL and MTB.

## Data and methods

### Patient data

A retrospective analysis was conducted on 49 cases of pathologically confirmed GL and 42 cases of MTB admitted between April 2019 and January 2022. All subjects signed informed consent. Neurological function assessment: Neurological function was assessed using the Mini-mental state examination (MMSE) scale. The MMSE scale has a total of 11 items, including orientation, memory, attention and calculation, discrimination, language. The total score is 0–30 points, with 27–30 points indicating normal cognitive function and <27 points indicating cognitive dysfunction.

### Eligibility criteria

Patients enrolled were all diagnosed with high-grade GL or MTB by pathological biopsy, aged >18 years old, and underwent a 3.0T MR examination before hospitalization, with the Glasgow Coma Scale (GCS) ≥9. Patients were excluded if they had severe consciousness or immune dysfunction, antibiotic drug use, surgery, radiotherapy, and chemotherapy in the first half year before admission, or liver and kidney dysfunction. Pregnant and lactating patients as well as referrals were also excluded. Exclusion criteria: extensive bleeding and necrosis in the lesion area; Image quality is poor; Allergic to contrast agents.

## Materials and methods

After fasting and water restriction for 2 h on the day of admission, all patients were examined by GE 1.5T MRI system. A 16-channel phased-array head coil was used, and the positioning line was located at the orbitomeatal baseline. The examination sequences used included T1WI, T2WI, FLAIR, and DWI. Layer thickness: 5 mm, spacing: 1 mm, FOV: 230 mm × 200 mm. T1WI: TR 450 ms, TE 15 ms. T2WI: TR 4,500 ms, TE 100 ms, FLAIR: TR 450 ms, TE 15 ms. The cubital vein was injected with gadopentetic acid dimeglumine salt injection (0.1 mmol/kg), a contrast agent, and the injection rate was 2 ml/s. The SE EPI sequence was adopted for DWI, and the relevant parameters were: layer thickness: 5 mm, layer spacing: 1 mm, TR: 7,300 ms, TE: 90 ms, FOV: 240 mm × 240 mm, diffusion sensitivity coefficient: 0, and 1,000 s/mm^2^.

### Outcome evaluation

All patients’ imaging results were reviewed and evaluated independently by two senior radiologists in our hospital. Imaging results were assessed using a single-blind method. A consensus was regarded as the final examination results, otherwise, a third imaging physician would be consulted. The original MRI images were uploaded to the workstation, and the regions of interest (ROI) were parenchyma, cystic, and peritumoral edema, and the areas of vascular, cystic, and necrosis were avoided. Fractional anisotropy (FA), apparent diffusion coefficient (ADC), and operative coefficient (K^trans^) were calculated. All measurements were made three times and the average value was taken.

### Statistics and methods

SPSS 22.0 performed statistical analysis of the data collected, and the comparison results with *P* < 0.05 were considered statistically significant. The data all meet the normal distribution, and the data all meet the condition of homogeneity of variance. The counting data (%) were compared by the chi-square test between groups. For measurement data ($\bar{\chi }$ ± s), the between-group and multi-group comparisons were made by independent samples *t*-test and one-way ANOVA plus LSD *post hoc* repeat test, respectively.

## Results

### Baseline data comparison

As shown in Table 1, patients with GL and MTB showed clinical comparability with no distinct difference in age, BMI, sex, smoking, drink alcohol, number of lesions, shape of the lesion, site of the lesion, and other baseline data (*P* > 0.05).

**TABLE 1:** Baseline data comparison.

	GL patients	MTB patients	*t* (or χ^2^)	*P*
Age (year)	63.997 ± 7.376	64.502 ± 6.616	0.338	0.736
BMI (kg/m^2^)	25.118 ± 3.105	24.679 ± 3.062	0.681	0.498
Sex			0.191	0.662
male/female	29/18	24/18		
Smoking			0.105	0.746
yes/no	24/23	20/22		
Drinking alcohol			0.035	0.851
yes/no	17/30	16/26		
Number of lesions			1.378	0.240
single/multiple	26/21	18/24		
Shape of the lesion			0.007	0.935
irregular/like round	7/40	6/36		
Site of the lesion			0.660	0.417
under the curtain/over the curtain	6/41	8/34		
Primary tumor			–	–
Lung cancer/breast cancer/colon cancer/esophagus cancer/stomach cancer	–	19/6/7/5/5		

### Comparison of clinical presentations between patients with glioma and metastatic tumor of the brain

Patients with GL mainly presented with neurological symptoms, including intracranial hypertension symptoms like dizziness, headache, and nausea, as well as intracranial localization symptoms like visual impairment and localized epilepsy, while hemiplegia was mostly seen in the later stage. MTB had basically the same clinical symptoms as GL, accompanied by symptoms of the primary tumor in some patients.

### Magnetic resonance imaging findings of glioma

**FIGURE 1:**
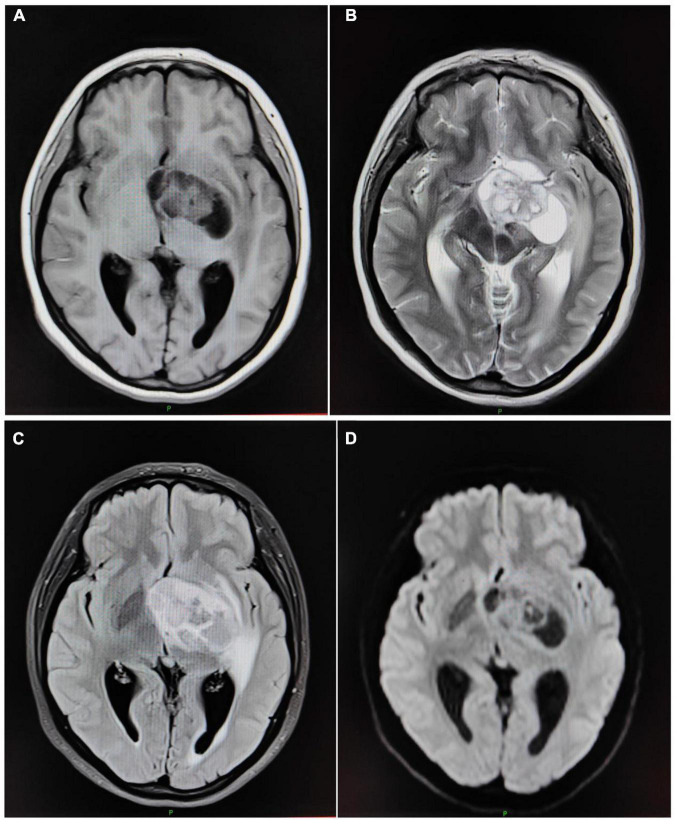
MRI findings of GL. The patient, a 62-year-old woman, presents with multiple, quasi-circular gliomas on the left side of the third lateral ventricle. **(A)** Transverse T1WI. **(B)** Transverse T2WI. **(C)** Transverse T2 FLAIR. **(D)** Transverse DWI.

The tumor parenchyma area of GL was mainly located in the tumor peripheral area and the cystic area was mainly located in the tumor central area. While the peritumor edema area was widely distributed, showing an irregular patchy edema zone. Contrast-enhanced scans showed that the tumor parenchyma was obviously enhanced with nodular and annular enhancement, while the tumor cystic and peritumor edema areas were not enhanced. As shown in Figure 1 and Table 2.

**TABLE 2:** MRI findings of GL.

	Parenchyma area	Cystic area	Peritumor edema area
T1WI	Isointensity/slightly low signal intensity	Low signal intensity	Slightly low signal intensity
T2WI	Slightly high signal intensity	Medium/high signal intensity	Isointensity/slightly low signal intensity
FLAIR	Isointensity/slightly high signal intensity	Low/slightly low signal intensity	Slightly high signal intensity
DWI	Isointensity/slightly high signal intensity	Slightly low signal intensity	Slightly low/isointensity signal intensity

### Magnetic resonance imaging manifestations of metastatic tumor of the brain

**FIGURE 2:**
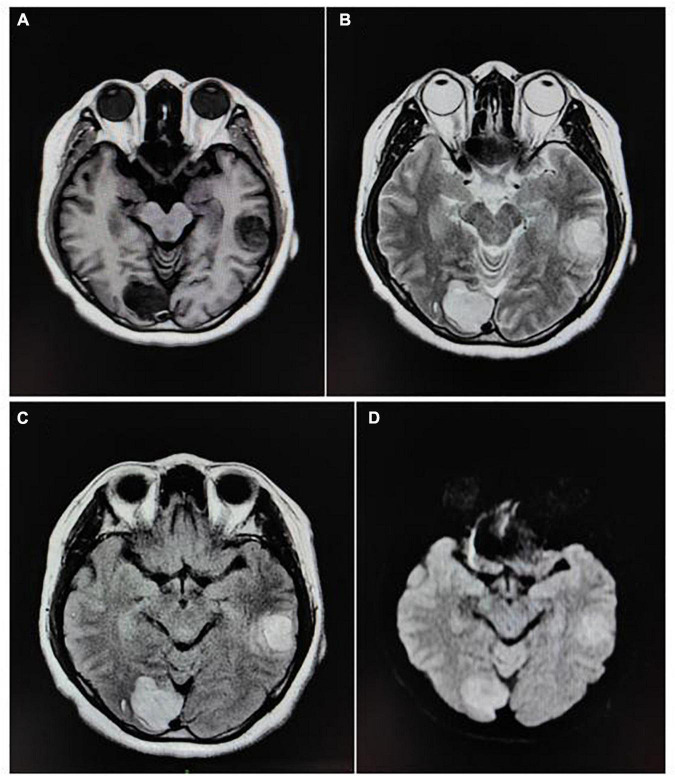
MRI results of MTB. The patient, a 57-year-old woman, presented with MTB in the left temporal lobe and right occipital lobe. The primary tumor was lung cancer, single and irregular. **(A)** Transverse T1WI. **(B)** Transverse T2WI. **(C)** Transverse T2 FLAIR. **(D)** Transverse DWI.

The distribution and status of tumor parenchyma, cystic, and peritumor edema areas of MTB were basically consistent with those of GL. As shown in Figure 2 and Table 3.

**TABLE 3:** MRI findings of MTB.

	Parenchyma area	Cystic area	Peritumor edema area
T1WI	Isointensity/slightly low signal intensity	Low signal intensity	Slightly low signal intensity
T2WI	Slightly high signal intensity	Medium/high signal intensity	Slightly high signal intensity
FLAIR	Isointensity/slightly high signal intensity	Low/slightly low signal intensity	Slightly high signal intensity
DWI	Isointensity/slightly high signal intensity	Slightly low signal intensity	Isointensity/slightly high signal intensity

### Comparison of fractional anisotropy values between patients with glioma and metastatic tumor of the brain

**FIGURE 3:**
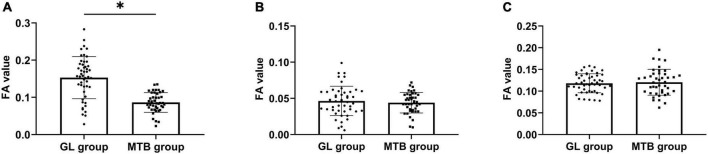
Comparison of FA values between patients with GL and MTB. **(A)** Comparison of FA values of the tumor parenchyma area. **(B)** Comparison of FA values of the tumor cystic area. **(C)** Comparison of FA values of the peritumor edema area, *P < 0.05.

Then, we compared the FA values between cases with GL and those with MTB in the same ROI. The results showed that there was no statistical difference in FA values of tumor cystic and peritumor edema areas between the two kinds of tumors (*P* > 0.05), but the FA values in the tumor parenchyma area of GL were higher (*P* < 0.05). From high to low, FA values in each ROI of patients with GL and MTB were the peritumor edema area, the parenchymal area, and the cystic area (*P* < 0.05). As shown in Figure 3.

### Comparison of apparent diffusion coefficient values between patients with glioma and metastatic tumor of the brain

**FIGURE 4:**
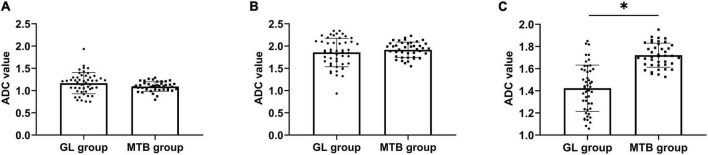
Comparison of ADC values between patients with GL and MTB. **(A)** Comparison of ADC values of the tumor parenchyma area. **(B)** Comparison of ADC values of the tumor cystic area. **(C)** Comparison of ADC values of the peritumor edema area, *P < 0.05.

There was no significant difference in ADC values in tumor parenchyma and cystic areas between GL and MTB, but the ADC values in the peritumor edema area of GL were lower (*P* < 0.05). From high to low, ADC values in each ROI of GL and MTB were tumor cystic area, peritumor edema area, and parenchymal area (*P* < 0.05). As shown in Figure 4.

### Comparison of K^trans^ values between patients with glioma and metastatic tumor of the brain

**FIGURE 5:**
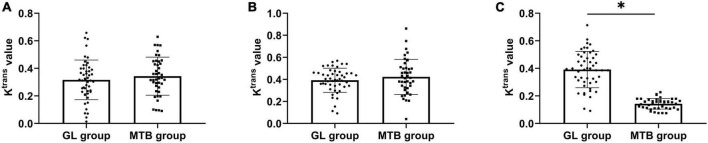
Comparison of K^trans^ values between patients with GL and MTB. **(A)** Comparison of K^trans^ values of the tumor parenchyma area. **(B)** Comparison of K^trans^ values of the tumor cystic area. **(C)** Comparison of K^trans^ values of the peritumor edema area, *P < 0.05.

No difference was found in K^trans^ in tumor parenchyma and cystic areas between GL and MTB (*P* > 0.05), but the K^trans^ in the peritumor edema area of GL was significantly higher compared with MTB (*P* < 0.05). The value of K^trans^ in each ROI of GL and MTB from high to low was tumor parenchyma area, cystic area, and peritumor edema area (*P* < 0.05). As shown in Figure 5.

### Association between magnetic resonance imaging findings and neurological function

**FIGURE 6:**
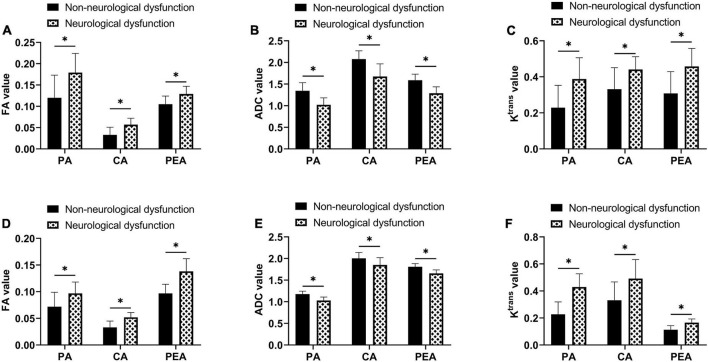
Association between MRI findings and neurological function. **(A)** Comparison of FA values of each region of interest between GL patients with and without neurological dysfunction. **(B)** Comparison of ADC values of each region of interest between GL patients with and without neurological dysfunction. **(C)** Comparison of K^trans^ values of each region of interest between GL patients with and without neurological dysfunction. **(D)** Comparison of FA values of each region of interest between MTB patients with and without neurological dysfunction. **(E)** Comparison of ADC values of each region of interest between MTB patients with and without neurological dysfunction. **(F)** Comparison of K^trans^ values of each region of interest between MTB patients with and without neurological dysfunction, *P < 0.05.

After evaluation by MMSE, 27 cases of patients with GL and 24 cases of patients with MTB were found to have neurological dysfunction. By comparison, it was found that in both patients with GL and MTB, the FA and K^trans^ values of all ROIs of cases with neurological dysfunction were higher than those without neurological dysfunction, while the ADC values were lower (*P* < 0.05). As shown in Figure 6.

## Discussion

Glioma, as the most common intracranial tumor, is a serious threat to patients’ life safety. Timely assessment and understanding of the occurrence and development of GL are of great significance to the life safety of patients (Przybylowski et al., 2021). How to effectively distinguish GL from MTB during contrast-enhanced MRI examination is the hotspot and difficulty of modern clinical research. In terms of imaging characteristics, both GL and MTB are manifested as circular or quasi-circular nodules or mass in plain and enhanced MRI images, as well as annular enhancement in contract-enhanced MRI scans, showing considerable similarity (Majigsuren et al., 2016). As far as the number of lesions is concerned, when both GL and MTB are single or multiple, the number of tumors is basically the same (Furuse et al., 2019). As for manifestations, MTB is also characterized by intracranial neurological symptoms rather than the primary tumor. In some cases of MTB, even the primary tumor cannot be found, which can only be diagnosed through pathological examination after surgery (Vallejo-Armenta et al., 2021). Such MTB can easily be misdiagnosed as GL, which seriously affects the timely and correct treatment of patients. In clinical practice, the effective and rapid reference criteria for distinguishing GL from MTB is lacking. This study is an extremely important reference tool for clinical practice.

In this study, we first found that the clinical manifestations of patients with GL and MTB included in this study were basically consistent with previous studies (Li et al., 2020; Tepe et al., 2021), that is, both the two kinds of tumors show neurological symptoms such as intracranial hypertension and intracranial localization. The only difference in MTB is that it will be accompanied by symptoms of the primary tumor, but there is still a lack of differentiation conditions in some primary tumors with strong concealment. Therefore, clinical symptoms alone cannot effectively distinguish GL from MTB. MRI is a spin imaging method based on the principle of nuclear magnetic resonance. According to the attenuation of the released energy in different structural environments inside the material, the emitted electromagnetic waves are detected by the gradient magnetic field, and the internal structural image of the object is drawn to indicate the location of the lesion and distinguish the nature of the lesion (Harisinghani et al., 2019). Therefore, we compared the contrast-enhanced MRI appearance of GL with MTB. The results showed that the examination results of tumor parenchyma area, cystic area, and peritumor edema area of the two tumors were basically the same, except for some certain differences in the peritumor edema area in T2WI and DWI sequences, which were also consistent with the results of previous studies (Jung et al., 2016; Jin et al., 2021). These studies can support our findings. This also suggests that the key to distinguishing GL from MTB lies in the peritumor edema area. Therefore, in the further comparison of relevant parameters in the ROIs of the two kinds of tumors, we found that the ADC value of the peritumoral edema region of GL was lower than that of MTB, while the values of K^trans^, V_e_, and V_p_ were all higher, with statistical significance. Reviewing previous studies, we found that MTB is simple angiogenic edema caused by hematogenous metastasis of a primary tumor outside the brain to the brain parenchyma, where the tumor parenchyma compresses the surrounding brain parenchyma and disrupts the blood–brain barrier (Mueller et al., 2020). The infiltration of GL into the surrounding brain parenchyma leads to the edema area around the tumor. In addition to simple angioedema, there are also a large number of tumor cells (Kim et al., 2020). Thus, water molecules around MTB are more easily dispersed than GL, resulting in an increase in ADC values. Similarly, it is precise because of the presence of tumor cells that GL mostly shows aggressive growth. Although the enhanced scan does not suggest an obvious enhancement, the perfusion volume and permeability in this area are higher, with new immature microvessels. The spatiality of cell activity is also greatly increased, thus contributing to the improvement of the K^trans^ value. Moreover, there are new immature blood vessels in the parenchyma and cystic areas of MTB, which also show high permeability, so the K^trans^ values of the two kinds of tumors do not show great differences. We believe that the increase of FA value in the parenchyma of GL is due to the fact that although both GL and MTB cause damage to nerve fiber bundles, but GL can also produce extracellular matrix. Extracellular matrix can adsorb and migrate tumor cells, resulting in higher FA values in parenchymal region than MTB (Tsiouris et al., 2019). At last, in the evaluation of the association between contrast-enhanced MRI findings and patients’ neurological function, we found that the FA and K^trans^ values in all ROIs of patients with neurological dysfunction were significantly higher compared with those without neurological dysfunction, while the ADC values were lower, which also suggest that contrast-enhanced MRI results also have a certain significance for evaluating patients’ neurological function and can provide more effective and objective suggestions for the future clinical treatment of GL or MTB. Contrast-enhanced MRI has achieved certain effects in early clinical screening, but the quality of images and data sources and other factors may lead to differences in results, which is still challenging to promote in clinical practice.

However, due to the limited number of cases included and the failure to consider the influence of pathophysiological characteristics of different primary diseases on the test results, a comparative study taking the above deficiencies in the later stage is warranted to further demonstrate the value of MRI in screening GL and MTB. This study only compared high-grade GL or MTB and did not compare low-grade GL and other brain tumor lesions, which need to be further studied in future. In addition, we will conduct a more in-depth and comprehensive experimental analysis on the evaluation value of MRI in intracranial tumors, so as to provide a more reliable reference for clinical practice.

In conclusion, contrast-enhanced MRI of the peritumor edema area can effectively distinguish GL from MTB, and improve the accuracy of early clinical screening, thus providing more reliable life security for patients.

## Data availability statement

The original contributions presented in the study are included in the article/supplementary material, further inquiries can be directed to the corresponding author.

## Ethics statement

The studies involving human participants were reviewed and approved by the Ethics Committee of our Hospital. The patients/participants provided their written informed consent to participate in this study.

## Author contributions

ZS wrote the manuscript. JJ designed the research. LX analyzed the data. XZ ensured the descriptions are accurate and agreed by all authors. All authors may have contributed in multiple roles.
